# Fact-finding survey of doctors at the departments of pediatrics and pediatric surgery on the transition of patients with childhood-onset chronic disease from pediatric to adult healthcare

**DOI:** 10.1371/journal.pone.0289927

**Published:** 2023-08-10

**Authors:** Ryota Kikuchi, Iori Sato, Yoichiro Hirata, Masahiko Sugiyama, Miwa Iwasaki, Hiromi Sekiguchi, Atsushi Sato, Seigo Suzuki, Mayumi Morisaki-Nakamura, Sachiko Kita, Akira Oka, Kiyoko Kamibeppu, Mari Ikeda, Motohiro Kato

**Affiliations:** 1 Division of Health Sciences and Nursing, Department of Family Nursing, Graduate School of Medicine, The University of Tokyo, Bunkyo-ku, Tokyo, Japan; 2 Department of Pediatrics, The University of Tokyo Hospital, Bunkyo-ku, Tokyo, Japan; 3 Department of Pediatric Surgery, The University of Tokyo Hospital, Bunkyo-ku, Tokyo, Japan; 4 Division of Nursing, The University of Tokyo Hospital, Bunkyo-ku, Tokyo, Japan; University of Sharjah, UNITED ARAB EMIRATES

## Abstract

**Background:**

The number of adult patients with childhood-onset chronic diseases is increasing. However, the process of transitioning these patients from child- to adult-centered medical services faces many difficulties. Despite the key role that doctors in the pediatric field are considered to play in transition, few fact-finding surveys about transition have been conducted among these doctors.

**Objective:**

The aim of this study was to demonstrate the current status and challenges in the transition of patients with childhood-onset chronic diseases by a fact-finding survey of pediatricians and pediatric surgeons at a university hospital.

**Methods:**

A cross-sectional survey was performed using an anonymous self-administered questionnaire. Seventy-six doctors of pediatrics and pediatric surgery (excluding junior residents) in a university hospital were asked to answer an anonymous self-report questionnaire. A multidisciplinary research team selected items related to the transitional process.

**Results:**

Sixty (79%) doctors participated, of whom 52 (87%) showed awareness of transition. No doctor answered that “Transition is conducted smoothly.” Doctors with shorter pediatric department experience had lower awareness and poorer experience with transition. In contrast to pediatric surgeons, pediatricians explained “job-seeking activities” and “contraceptive methods” to the patient, and reported a higher patient age at which to initiate explanation of transition to the patient and his/her family. Among factors inhibiting transition, 39 (65%) respondents selected “The patient’s family members do not desire transition” and 34 (57%) selected “Although a relevant adult healthcare department is available, it will not accept the patient.” The medical providers most frequently considered to have responsibility for playing a central role in the transition process were “pediatrician/pediatric surgeon,” “medical social worker,” and “regional medical liaison office.”

**Discussion:**

To promote transition, pediatric and adult healthcare departments should share concerns about and cooperate in the establishment of more effective methods of transition, and provide multidisciplinary collaboration to support patients and their families.

## Introduction

Thanks to the improved prognosis of childhood-onset chronic diseases [1–7], the number of pediatric patients with chronic diseases reaching adulthood is expected to increase in the future [8–10]. For example, the number of patients with congenital heart disease reaching adulthood has increased by more than 50% over the 10-year period to 2010 [11]. One result is that the medical care of these patients has recently become a major issue. These patients have been referred to as, for example, “youth with special healthcare needs” [12] or “adolescents and emerging adults with special healthcare needs,” [13]. They have diverse medical conditions, including the continuation of therapy for the underlying disease, complications of therapies received in childhood, and the occurrence of new conditions in adulthood [14–18].

The continuation of treatment in pediatric patients with chronic diseases who will or have already attained adulthood within the framework of pediatric healthcare services is challenging, and these patients therefore require transfer to adult medical care departments. Specifically, they require support to facilitate transfer from the child-centered to the adult-centered healthcare system, and to assume central responsibility for their own disease management and decision-making, a role previously filled by their caregivers [19, 20]. In general, transition is defined as the purposeful, planned movement of adolescents and young adults with chronic physical and medical conditions from child-centered to adult-oriented health-care systems [21]. It is desirable that these transitions be performed in a stepwise and planned manner so that patients receive appropriate services not only for their medical needs but also for their growth and development [5]. From this clinical need, the establishment of transition support programs is now being promoted in Japan [5, 22, 23].

Nevertheless, the promotion of transition is complex. Factors affecting transition include those associated with pediatric healthcare providers, those associated with adult healthcare providers, and those associated with the patients and their family members (primarily caregivers) [4]. Importantly, the establishment of transition support programs should be consistent with the medical characteristics of the patients, the characteristics and status of their medical institution, and the social background and healthcare system. Establishing this type of program also requires detailed investigation of the awareness and recognition of transition among pediatric healthcare providers, especially medical doctors belonging to pediatric healthcare departments (i.e., departments of pediatrics and pediatric surgery).

Pediatricians and pediatric surgeons play a central role in transitional care. How do pediatricians and pediatric surgeons support transitions and what difficulties do they have? Few studies have answered this research question. There are multiple stakeholders in transition (i.e., patients, parents, pediatric providers, adult providers [24]). Research on transition began around 1990 [13], and many studies have assumed that the subjects of the investigation are the patients themselves and their parents or caregivers. Studies have shown that patients experience changes during the transition period, including changes in important relationships they established during pediatric care, adjustment to unfamiliar hospital wards, and responsibility for self-management [25]. Furthermore, based on the research being conducted on transitional support in various diseases [1, 16, 24, 26, 27], transitional support is considered to be a highly needed topic. In a study of parents, parents were found to have concerns about delegating responsibility to their children, albeit that they viewed transition in a positive light [28]. To date, however, few studies have been conducted among healthcare providers, and those conducted with nurses have revealed differences in attitudes toward transitional support between nurses in the pediatric and adult department [29]. Despite the general consideration that doctors in the pediatric field play a key role in transition, few fact-finding surveys about transition among such doctors have been conducted. Ishizaki et al.’s (2012) survey of pediatricians at children’s hospitals and related medical institutions and nurses at pediatric departments found that a marked majority of pediatricians considered transitional support programs necessary and were positive about transition to adult healthcare departments, but noted that networking between pediatric and adult healthcare services was insufficient [30]. Mitsuya et al. (2014) surveyed medical staff in the pediatric ward of a general perinatal medical center and reported that the medical doctors considered transitional support programs necessary, but felt that there were no adult healthcare departments which could accept patients with childhood-onset chronic diseases [31]. However, these surveys did not address certain transition practices, including the patients’ age at which doctors at pediatric healthcare departments should begin to explain transition to the patients and/or their family members, the method and content of such explanation, and the type of provider who should play a central role in the transitional process. In addition, factors of awareness and recognition specific to university hospitals may require consideration independently of other types of institution.

Based on this background, the aim of this study was to demonstrate the current status of and challenges in the transition of patients with childhood-onset chronic diseases among pediatricians and pediatric surgeons at a university hospital. Specifically, we conducted a fact-finding survey of pediatricians and pediatric surgeons at a university hospital regarding the current status of and challenges in the transition of patients with childhood-onset chronic diseases, and how transition support programs should be operated. This study was expected to provide suggestions for the establishment of a transitional support program.

## Methods

### Research design

A cross-sectional survey was conducted using an anonymous self-administered questionnaire. A cross-sectional study design was used to gain an overall picture of how transitional support is provided in the hospital. Medical doctors working at the Departments of Pediatrics and Pediatric Surgery at the university hospital were eligible to participate. Junior residents were excluded from the survey. The number of eligible medical doctors was 76, consisting of 65 (85.5%) pediatricians and 11 (14.5%) pediatric surgeons.

### Study setting

The university hospital where the survey was conducted is located in Tokyo and is one of the leading general hospitals in Japan. The hospital has approximately 40 departments, including adult departments. There are approximately 1,100 beds, with an average of 1,100 inpatients per day and 3,000 outpatients per day. The hospital has many adult departments, but only a few cases are being transitioned. The pediatrics and pediatric surgery departments work together to provide medical care to patients with serious illnesses, mainly from the neonatal period to adolescence. For the purposes of this study, the departments of pediatrics and pediatric surgery were included in the study.

### Survey administration and collection

Member doctors of the Task Force for Establishment of a Transition Outpatient Clinic distributed a written explanation of the survey, the questionnaire, and a return envelope to eligible survey participants. After reading the written explanation, those who consented to participate in the survey answered the questionnaire and placed their response in the return envelope. The envelopes were collected at places designated by each department (i.e., separately for pediatricians and pediatric surgeons). No incentive to participate was provided. The survey was conducted in August and September, 2015.

### Ethical considerations

This survey was approved by the Ethics Board of the Graduate School of Medicine, The University of Tokyo (Approval Number 10942). A written explanation of the survey provided to all eligible participants clearly stated that participation was voluntary and that they would not be disadvantaged by declining to participate. Consent forms were not obtained from study participants, as the questionnaire was conducted anonymously. To prevent identification of the participants, the survey did not ask about the sub-specialty group (e.g., cardiology, hematology-oncology, neurology, digestive surgery) to which the participant belonged. Return of a completed questionnaire was deemed to indicate consent to participate.

### Questionnaire

The framework for survey items was established after due discussion by a multidisciplinary research team—the Task Force for Establishment of a Transition Outpatient Clinic—at the medical institution. The questionnaire items were determined with reference to previous studies [25, 30–40], and took account of the characteristics of university hospitals and the present status of the subject medical institution.

Regarding demographic characteristics, the participant’s department (i.e. Pediatrics or Pediatric Surgery), years of experience as a medical doctor, and years of engagement in the pediatric field were surveyed. The questionnaire consisted of 24 items. Response format was mainly multiple-choice, with free-response questions as appropriate.

The current status of transition was assessed by survey questions asking participants about transition (*“How much do you know about the transition*?*”*), experience of involvement in transition (*“To what extent have you been involved in transition*?*”*), degree of smoothness of transition (*“Do you think transition is conducted smoothly*?*”*), age at which transition is explained to the patient, issues specific to adulthood which are explained to the patient, and how disease conditions are explained to the patient and his/her family members.

Regarding challenges in transition, the following topics were explored: factors inhibiting transition, factors facilitating transition, information which medical doctors should explain to the patient and/or his/her family members on transfer to a relevant adult healthcare department, types of medical provider who should play a central role in the transition process, upper patient age limit for attendance at the pediatric department by department, patient age at which an explanation of transition should be initiated with the patient, and patient age at which an explanation of transition should be initiated with the patient’s family.

### Data analysis

All returned questionnaires were to be analyzed, excluding any missing values. Descriptive statistics were calculated for all variables. For continuous variables, mean and standard deviation were calculated. For discrete variables, frequency and proportion were calculated. Number of years as a medical doctor and years of engagement in the pediatric field were each classified into the three categories of less than 10 years, 10 to 19 years, and over 20 years. The variables were aggregated consistently with their characteristics by years of experience or clinical department.

## Results

### Participant characteristics

Of 76 eligible medical doctors, 60 (50 pediatricians, 10 pediatric surgeons) agreed to participated in the survey (response rate: 78.9%). Demographic characteristics of participants are shown in Table 1. Mean years of experience as a medical doctor was 15.4 years: 16 (26.7%) had less than 10 years, 30 (50.0%) had 10 to 19 years, and 14 (23.3%) had over 20 years in total. Mean years of engagement in the pediatric field was 13.1 years: 23 (39.7%) had less than 10 years, 22 (37.9%) had 10 to 19 years, and 13 (22.4%) had over 20 years in total.

**Table 1:** Demographic characteristics of participants.

	Total (N = 60)		Department of Pediatrics (n = 50)		Department of Pediatric Surgery (n = 10)	
(years)	mean ± s.d. or n (%)	Range	mean ± s.d. or n (%)	Range	mean ± s.d. or n (%)	Range
Years as a medical doctor	15.4 ± 8.8	2.4–39.3	15.6 ± 9.3	2.4–39.3	14.5 ± 6.7	6.3–28.2
<10	16 (26.7)		14 (28.0)		2 (20.0)	
10 to 19	30 (50.0)		24 (48.0)		6 (60.0)	
≥ 20	14 (23.3)		12 (24.0)		2 (20.0)	
Years of experience in the pediatric field*	13.1 ± 9.1	0.8–39.3	13.8 ± 9.4	0.8–39.3	9.4 ± 6.6	2.3–24.9
<10	23 (39.7)		17 (34.7)		6 (66.7)	
10 to 19	22 (37.9)		20 (40.8)		2 (22.2)	
≥ 20	13 (22.4)		12 (24.5)		1 (11.1)	

* N = 58 (n = 49 in the Department of Pediatrics and n = 9 in the Department of Pediatric Surgery).

### Current status of transition

**Fig 1:**
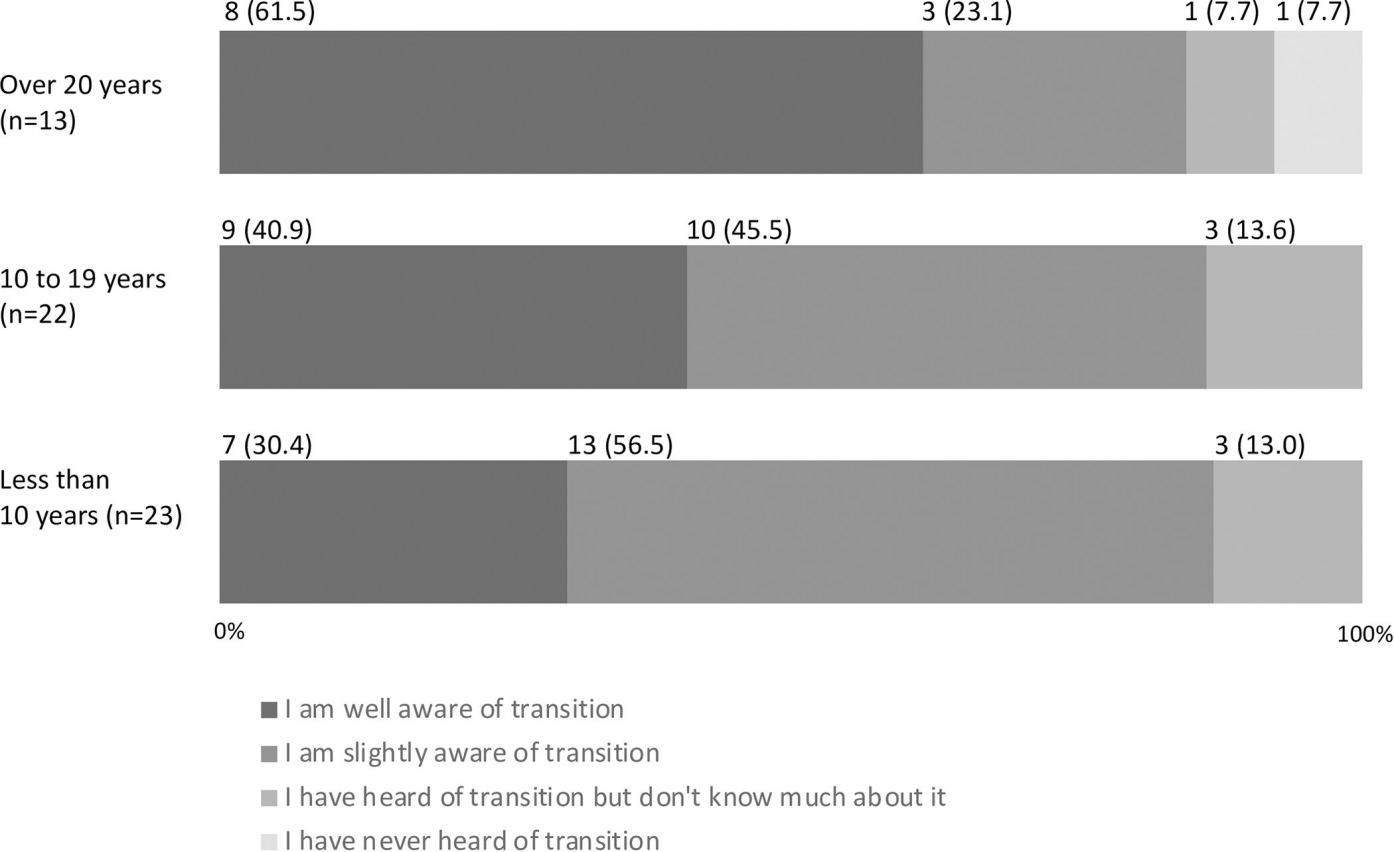
Degree of awareness about transition by years of engagement in the pediatric field (n (%), N = 58).

**Fig 2:**
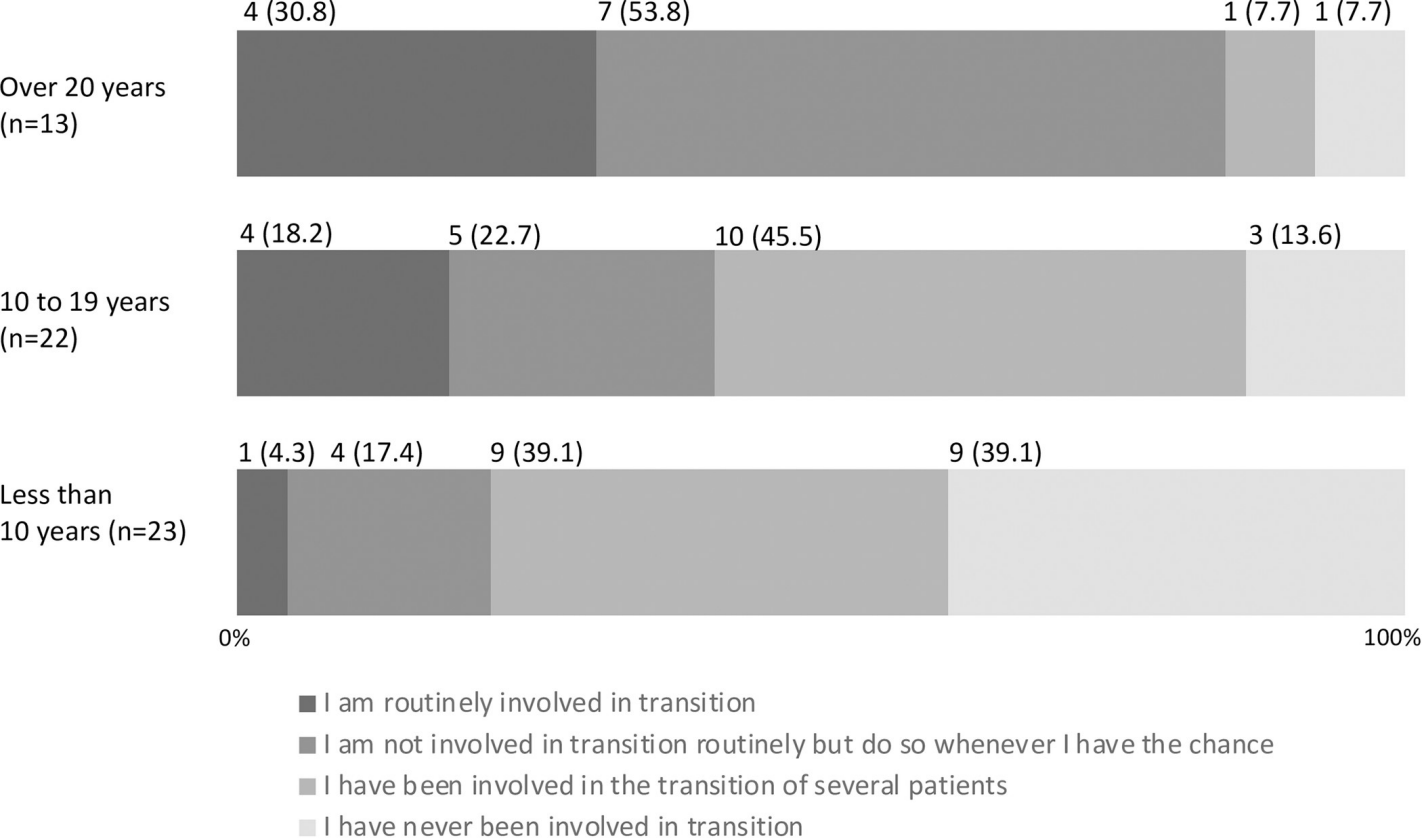
Experience of involvement in transition by years of engagement in the pediatric field (n (%), N = 58).

**Fig 3:**
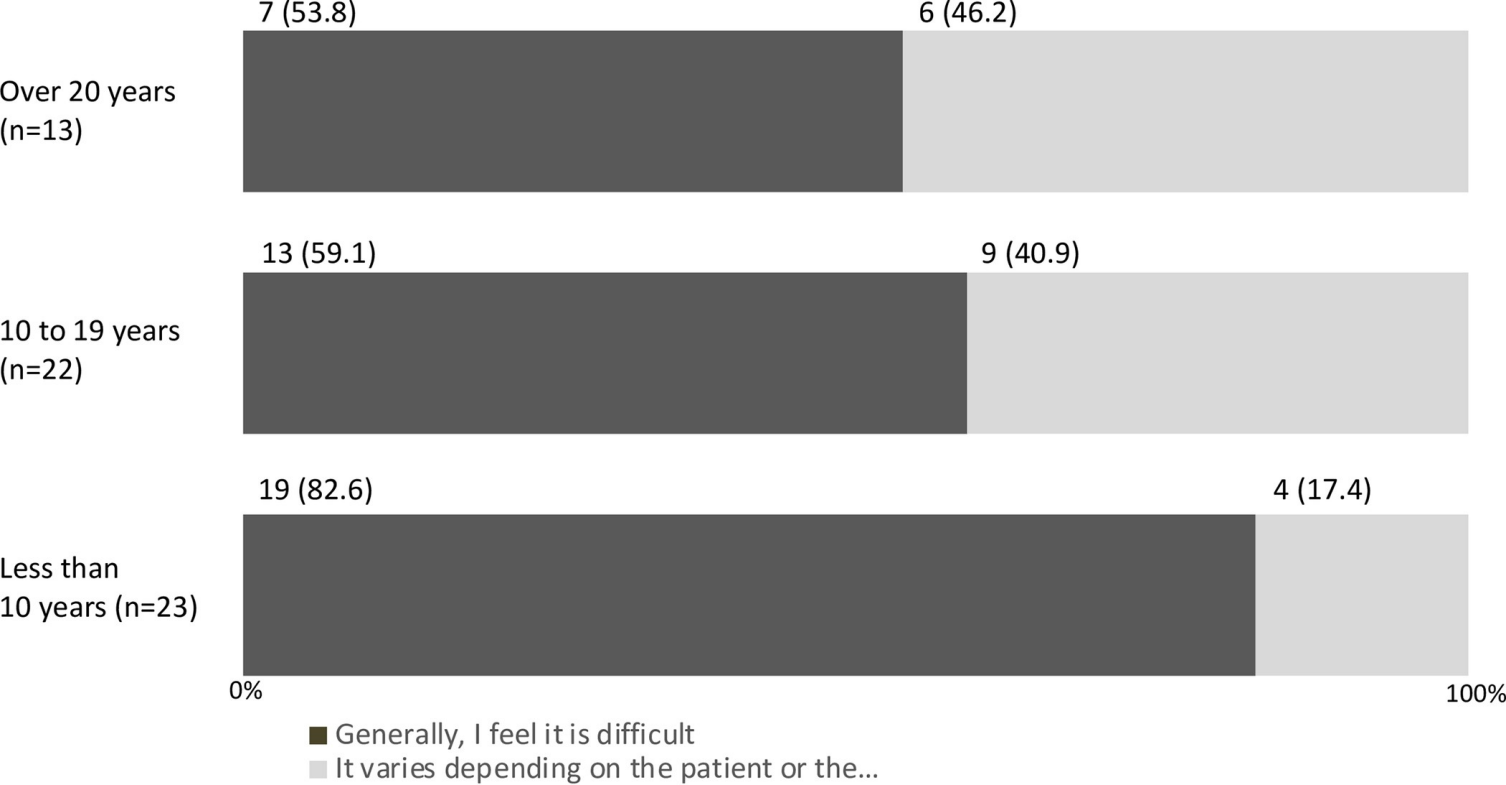
Degree of smoothness of transition by years of engagement in the pediatric field (n (%), N = 58).

A total of 52 (86.7%) responders showed awareness about transition (i.e., “I am well aware of transition” and “I am slightly aware of transition”), and 47 (78.3%) had experience of being engaged in transition (i.e., other than “I have never been involved in transition”) (Table 2). None of the responders thought that transition was smooth (Table 2). Responders who with a shorter engagement period in the pediatric field tended to have lower awareness of transition (Fig 1), had scarce experience of involvement in transition (Fig 2), and had difficulty in transition (Fig 3).

**Table 2:** Current status and challenges in transition.

		Total (N = 60)	Department of Pediatrics (n = 50)	Department of Pediatric Surgery (n = 10)
		n (%)	n (%)	n (%)
**Current status of transition**				
Degree of awareness about transition	I am well aware of transition	25 (41.7)	21 (42.0)	4 (40.0)
	I am slightly aware of transition	27 (45.0)	22 (44.0)	5 (50.0)
	I have heard of transition but don’t know much about it	7 (11.7)	6 (12.0)	1 (10.0)
	I have never heard of transition	1 (1.7)	1 (2.0)	0 (0)
Experience of involvement in transition	I am routinely involved in transition	9 (15.0)	8 (16.0)	1 (10.0)
	I am not involved in transition routinely but do so whenever I have the chance	16 (26.7)	15 (30.0)	1 (10.0)
	I have been involved in the transition of several patients	22 (36.6)	17 (34.0)	5 (50.0)
	I have never been involved in transition	13 (21.7)	10 (20.0)	3 (30.0)
Degree of smoothness of transition	Generally, I feel it difficult	40 (66.7)	30 (60.0)	10 (100)
	It varies depending on the patient or the disease	20 (33.3)	20 (40.0)	0 (0)
	Generally, I feel it is smooth	0 (0)	0 (0)	0 (0)
Age at which to explain transition to the patient* (Multiple answers allowed)	If the patient doesn’t ask me beforehand, I explain when they reach a certain age	39 (83.0)	34 (68.0)	5 (50.0)
	I explain when the patient asks me	7 (14.9)	5 (10.0)	2 (20.0)
	I explain to patients when I first meet them	4 (8.5)	4 (8.0)	0 (0)
	I do not give explanations	0 (0)	0 (0)	0 (0)
Issues specific to adulthood which are explained to the patient (Multiple answers allowed)	Pregnancy/giving birth	34 (56.7)	29 (58.0)	5 (50.0)
	Diseases in adulthood such as lifestyle-related diseases and cancer	30 (50.0)	26 (52.0)	4 (40.0)
	Going to the next stage of education	29 (48.3)	27 (54.0)	2 (20.0)
	Job-seeking activities	19 (31.7)	19 (38.0)	0 (0)
	Health care systems available in adulthood	15 (25.0)	14 (28.0)	1 (10.0)
	Contraceptive methods	6 (10.0)	6 (12.0)	0 (0)
	Others	3 (5.0)	3 (6.0)	0 (0)
How disease conditions are explained to the patient and his/her family members	Both the patient and his/her family members frequently attend explanation meetings, but depending on the situation, the explanation is given to them separately	46 (76.7)	37 (74.0)	9 (90.0)
	Both the patient and his/her family members always attend explanation meetings	8 (13.3)	8 (16.0)	0 (0)
	The explanation is frequently given to the patient and his/her family members separately, but sometimes both attend the meeting, depending on the situation	6 (10.0)	5 (10.0)	1 (10.0)
	The explanation is always given to the patient and his/her family members separately	0 (0)	0 (0)	0 (0)
**Challenges for transition**				
	Understanding of transition in the relevant adult healthcare department	48 (80.0)	40 (80.0)	8 (80.0)
	Presence of a department or a person in charge which is responsible for selecting the medical institution to which the patient will be transferred and communicating with that institution	34 (56.7)	27 (54.0)	8 (70.0)
	Patient and/or his/her caregivers change their mindset	33 (55.0)	26 (52.0)	7 (70.0)
	Clarify the pediatric department and the corresponding adult department	27 (45.0)	21 (42.0)	6 (60.0)
	Availability of information about the medical institution to which the patient will be transferred (if such information is available, then I myself can address the transition)	23 (38.3)	22 (44.0)	1 (10.0)
	Others	5 (8.3)	4 (80.0)	1 (10.0)
Information which should be explained by medical doctors to the patient and/or his/her family members when transferring to a relevant adult healthcare department (Multiple answers allowed)	Knowledge about the disease and/or symptoms	57 (95.0)	47 (94.0)	10 (100)
	Objectives and details of therapy	44 (73.3)	37 (74.0)	7 (70.0)
	Knowledge of complications	43 (71.7)	36 (72.0)	7 (70.0)
	Issues specific to adulthood	42 (70.0)	37 (74.0)	5 (50.0)
	How to take responsive actions when physical condition is not good	40 (66.7)	32 (64.0)	8 (80.0)
	How to visit a doctor and/or a medical institution	31 (51.7)	26 (52.0)	5 (50.0)
	Self-management of medications	30 (50.0)	27 (54.0)	3 (30.0)
	Self-management of information about diagnosis and treatment (e.g., clinical laboratory data)	27 (45.0)	20 (40.0)	7 (70.0)
	How to explain their disease and its issues to others	23 (38.3)	20 (40.0)	3 (30.0)
	Others	2 (3.3)	1 (2.0)	1 (10.0)
Types of medical provider who should play a central role in the transition process (Multiple answers allowed)	Pediatrician/pediatric surgeon	36 (60.0)	32 (64.0)	4 (40.0)
	Medical social worker	13 (21.7)	9 (18.0)	4 (40.0)
	Regional medical liaison office	12 (20.0)	9 (18.0)	3 (30.0)
	Medical doctor in an adult healthcare department	5 (8.3)	4 (8.0)	1 (10.0)
	Nurse	5 (8.3)	5 (8.3)	0 (0)
	Clinical psychologist	1 (1.7)	1 (1.7)	0 (0)
	Pharmacist	0 (0)	0 (0)	0 (0)
	Others	1 (1.7)	1 (2.0)	0 (0)

* This question was posed to the 47 respondents who chose the following answers regarding their experience of being involved in transition: "I am routinely involved in transition," "I am not involved in transition routinely but do so whenever I have the chance," and "I have been involved in the transition of several patients."

Regarding age at which they explain transition to the patient, 39 (83.0% from 47 respondents) respondents selected “if the patient doesn’t ask me beforehand, I explain when they reach a certain age.” “I explain to patients when I first meet them” was selected by pediatricians only. Regarding issues specific to adulthood which are explained to the patient, the most reported responses were “pregnancy/giving birth” by 34 (56.7%) respondents, and “diseases in adulthood such as lifestyle-related diseases and cancer” by 30 (50.0%) respondents. “Job-seeking activities” and “contraceptive methods” were selected by pediatricians only. Typical examples of “others” were “obtaining a driver’s license” and “nutrition management.”

### Challenges for transition

**Fig 4:**
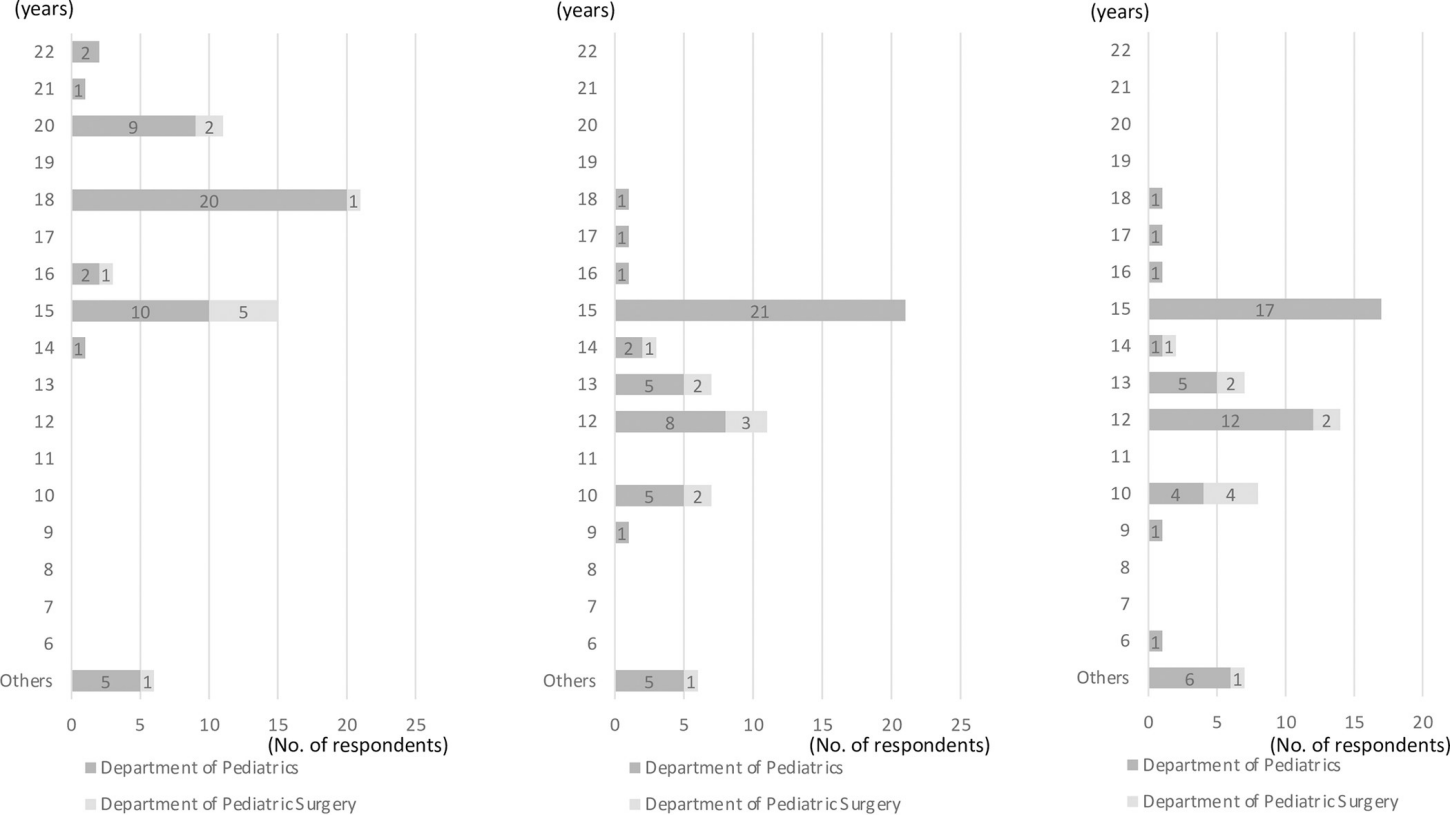
1. Upper patient age limit to attend the pediatric department by department (N = 60), 2. Patient age for initiation of explanation of transition to the patient (N = 59), 3. Patient age for initiation of explanation of transition to the patient’s family (N = 60).

The most frequently reported factors inhibiting transition were “The patient’s family members do not desire transition” by 39 (65.0%); “Although a relevant adult healthcare department is available, it will not accept the patient” by 34 (56.7%); “The patient him/herself does not desire the transition” by 32 (53.3%) (Table 2). Typical examples of “others” were “pediatrics is too kind” and “there is no adult department that can treat overlapping diseases like pediatrics.” The most frequently reported factors facilitating transition were as follows: “understanding of transition to the relevant adult healthcare department” by 48 (80.0%), “the presence of a department or a person in charge which is responsible for selecting a medical institution to which the patient will be transferred and communicating with that institution” by 34 (56.7%), “the patient and/or his/her caregivers change their mindset” by 33 (55.0%) (Table 2). Typical examples of “others” were “to create a successful transition model” and “to establish a period for parallel examinations in pediatrics and adult clinical departments.” The most reported information which should be explained by medical doctors to the patient and/or his/her family members when transferring to a relevant adult healthcare department were “knowledge about the disease and/or symptoms,” “objectives and details of therapy,” “knowledge of complications,” and “issues specific to adulthood” (Table 2). The most reported types of medical provider who should play a central role in the transition process was “pediatrician/pediatric surgeon,” “medical social worker,” and “regional medical liaison office” (Table 2). The most reported upper patient age limit to attend pediatric department by department was 18 years (Fig 4 (1)). The most reported patient age for initiation of explanation about transition to the patient was 15 years (Fig 4 (2)). The most reported patient age for initiation of explanation about transition to the patient’s family members was 15 years (Fig 4 (3)). Generally, pediatricians reported age was wider than pediatric surgeons.

## Discussion

This research was conducted as a fact-finding survey about transition among pediatricians and pediatric surgeons working at a university hospital. The main results were that none of the doctors felt that transition occurred smoothly; doctors with shorter experience in the pediatric field had lower awareness of and less experience with transition; and doctors thought that greater understanding of the patient, his/her family, and adult healthcare department was necessary for successful transition. These findings suggest that pediatric and adult healthcare departments should share concerns about and cooperate in the establishment of more effective methods of transition, and provide support for patients and their families through multidisciplinary collaboration.

This survey has provided some novel findings. The first is the different attitude toward transition between pediatricians and pediatric surgeons. Regarding issues specific to adulthood that are explained to the patient, only pediatricians chose the answers of “Job-seeking activities” and “Contraceptive methods” while no pediatric surgeons chose them. Regarding the age at which it is desirable for patients to receive clinical management at a pediatric department and the age at which to initiate explanation of transition to the relevant adult healthcare department, many doctors at the pediatrics department answered “15 years,” but all pediatric surgeons answered 10 to 14 years. This age is comparable to the results obtained from previous studies: the most frequent answer in the survey by Mitsuya et al. (2014) was junior high school students (typically 12–14 years of age) [31], while a review by Fegran et al. (2014) reported initiation of explaining transition to the patients at age 14 years [25]. In the present survey, pediatricians chose higher ages than pediatric surgeons, which may serve to indicate that patient age for this variable is affected by the differences among patients treated at different departments in terms of disease type, length of period for treatment and clinical management, and other relevant matters. Adolescence is in itself a transitional period from childhood to adulthood, a period of role change in their life [41]. Accordingly, these differences between pediatrics and pediatric surgery should be taken into account when establishing transition support programs.

The second new finding of this survey is that the pediatricians and pediatric surgeons recognized that the other professions they considered suitable for transitional support are social workers and regional medical liaison offices. These social resources may be expected to act as a bridge between medical doctors in pediatric and adult healthcare departments and patients and their family members. In this study, the most frequently selected factor inhibiting transition was “The patient’s family members do not desire the transition,” also suggested by the systematic reviews [42, 43]. The most frequent for factors facilitating transition was “Understanding of transition to the relevant adult healthcare department.” These results suggest that facilitating transition involves multiple factors. Since doctors in the pediatric field consider that they themselves should play a central role in the transition process, it is important that doctors in the pediatric field should establish good relations with those in adult healthcare services as well as with patients and their family members. Consistent with this, the survey by Mitsuya et al. (2014) [31] found that the answer most frequently selected by pediatricians as inhibiting transition among patient and family members was “The patient’s family members refuse the transition.” In their survey, however, the most frequently chosen answer for inhibitory factors on the hospital side was “There are only a small number of medical institutions or departments which will accept the patient concerned.” This difference may be attributable to differences in the characteristics of the two medical institutions surveyed: Mitsuya et al. conducted their survey at a general perinatal medical center [31], whereas the current survey was performed at a university hospital. Considering these results, promotion of transition should require not merely the presence of adult healthcare departments, but also their understanding and acceptance of transitioning patients. The importance of establishing effective communication and networking between pediatric and adult healthcare departments has been noted [15, 30, 44, 45]. At our hospital, we first need to establish a system on the provider side. However, building a foundation for collaboration between pediatric and adult healthcare departments is not easy. Bell et al. (2011) pointed out the importance of having common understanding on what is mutually expected and what should be included in the transition protocol between the pediatric and the adult healthcare. [15]. They emphasized that the pediatric health care providers need to communicate well not only with the adult counterparts, but also with the patients and their family, both in written information (medical records) and in-person meetings [15]. Previous studies suggested the effectiveness of well-structured interventions for transition [46, 47]. In the present survey, regarding the types of providers who should play a central role in the transition process, those engaged in bridging with social resources such as social workers and regional medical liaison officers were the second most frequently selected answers, following “pediatrician/pediatric surgeon.” It has been reported that patients have questions and anxieties about healthcare insurance coverage when they reach adulthood [12, 15]. Systematic reviews suggest that transition coordinators play an effective role in interventions for transition [48, 49]. The present results suggest that promoting collaboration through the above types of providers may reinforce the in-house system which supports transitions.

The survey results indicated that a substantial majority of medical doctors who engaged in pediatric healthcare know and experience transition, and that the doctors feel that transitions were difficult to perform. The level of awareness and experience vary depending on years of experience in the pediatric field. Shorter experience was associated with a lower level of awareness and experience of transition. These results may be partially attributable to the fact that the shorter the years of experience in the pediatric field, the fewer the opportunities to treat patients in the transition stage.

When evaluating the results of this research, it is important to consider the characteristics of the subject medical institution, a university hospital with a total of 40 departments for both pediatric and adult healthcare services which provides specialized medical care over a wide range of diseases. Patients who visit this hospital tend to suffer from more serious diseases than those who go to general hospitals, and to have rare diseases. In addition, the level of specialty in each department of this hospital is markedly high. These characteristics of both patients and the hospital may influence recognition of the patient age at which transition should be initiated.

Based on these results, some recommendations can be made for future interventions and future directions. First, to accelerate learning process of doctors about transition, it may therefore be useful to first hold educational meetings about transition with the aim of deepening recognition and understanding of this practice. Second, it may be important to conduct clinical case conferences in a manner specific to departments or sub-specialty, such as the pediatric cardiovascular group or pediatric oncology group. The aim of such case conferences should be to share experience of transitional care among participants and to establish a framework which facilitates transition on an organization-wide basis. Third, since the survey was conducted in a single hospital, different results might be obtained if the survey were conducted with the inclusion of other hospitals. Finally, a survey of doctors at adult healthcare departments is required.

This study has several limitations. First, this survey was conducted anonymously, which might have caused selection bias. Although the response rate was high, the doctors who responded to this survey might have been more interested in the issues related to transition than those who did not. Gender and race/ethnicity were not examined in this study to prevent participant identification, but we have not been able to examine the possibility that these characteristics may have played a role in the results. Second, the survey was conducted at a university hospital and, due to the nature of its clinical setting, the pediatricians and pediatric surgeons at this hospital may differ from those at other types of medical institution with regard to their recognition of and experience with transitions. For example, pediatric patients who visit the hospital typically have relatively severe disease and required lifelong treatment; accordingly, the doctors might explain to them not only their illnesses, symptoms, and treatment, but also pregnancy childbirth and job-seeking activities. Due to the characteristics of the patients who attend, these results may differ in municipal hospitals and clinics. Third, the survey did not ask about the clinical management group to which the participants belonged. This prevented evaluation of differences in transition by disease type. Fourth, there is generalizability related to medical insurance. In many cases in Japan, public medical insurance provides seamless medical care from pediatric to adult care, so this study did not investigate medical insurance as a barrier to transition. The results of this study may differ from those in other countries. Fifth, the validity and reliability of the questionnaire has not been tested and may have missing elements. Finally, because of the small sample size, we analyzed data by comparison with descriptive statistics only and did not conduct statistical testing. Since this study revealed the diversity of issues that need to be addressed in transitional support, future research using a qualitative design is warranted.

This research was performed among medical doctors working at pediatric healthcare departments. The actual status of transition and its awareness by doctors may differ between pediatric and adult healthcare departments. The Task Force for Establishment of a Transition Outpatient Clinic is currently performing a fact-finding survey of transition among nurses belonging to pediatric and adult healthcare departments; and to best use the findings obtained, the Task Force is currently organizing an in-house system to establish a transition outpatient clinic.

## Conclusions

The pediatricians and pediatric surgeons in this survey reported difficulty with transition. Doctors with shorter experience in the pediatric field had lower awareness of and less experience with transition. There were differences between pediatrics and pediatric surgery in the content to be explained to patients and the starting age of explanations regarding the transition to patients and their families. Further, the doctors thought that successful transition requires the understanding of the patient, his/her family, and the adult healthcare department. To promote transition, it is necessary for the pediatric and adult healthcare departments to share concerns about and cooperate in the establishment of more effective methods of transition, and provide support for patients and their families through multidisciplinary collaboration.
